# Stabile Subordinationsverhältnisse. Aufstieg und Niedergang des Mittelbaus in der Hochschulmedizin der 1960er Jahre

**DOI:** 10.1007/s00048-024-00392-3

**Published:** 2024-07-10

**Authors:** Heiko Stoff

**Affiliations:** Institut für Ethik, Geschichte und Philosophie der Medizin, Medizinische Hochschule Hannover, Carl-Neuberg-Str. 1, 30625 Hannover, Deutschland

Im Sommer 1961 initiierte der Deutsche Wissenschaftsrat die Gründung Medizinischer Akademien. Ausdrückliches Ziel war die Ablösung der hierarchisch strukturierten Medizinischen Fakultät durch ein auf einem starken Mittelbau beruhendes Departmentsystem nach US-amerikanischem Vorbild (Paulus 2010: 393–411). Das Kollektivum „Mittelbau“ wurde in Westdeutschland überhaupt erst in diesem Zusammenhang gebräuchlich, um die große akademische Gruppe der graduierten „Nicht-Ordinarien“ zu bezeichnen, der bei den Neugründungen von Hochschulen eine zentrale Rolle zukommen sollte. Diese kurze Blütezeit des Mittelbaus endete jedoch bereits wieder Mitte der 1970er Jahre, als in den Hochschulgesetzgebungen – trotz gewisser Zugeständnisse bei der Mitbestimmung – die Vorrangstellung der Ordinarien als „Professorenmehrheit“ verbindlich festgeschrieben wurde. Der Mittelbau wurde damit zugleich wieder von der Lösung zum Problem herabgestuft. Aufstieg und Fall des Mittelbaus in der westdeutschen Universitätsmedizin werden im Folgenden kursorisch skizziert.

## Der Plan: Teamwork im Departmentsystem

Im September 1957 war der Deutsche Wissenschaftsrat mit dem ausdrücklichen Ziel gegründet worden, Ideen für eine Hochschulreform zu entwickeln (Bartz 2007). Der Medizin kam dabei eine Sonder- und Schlüsselrolle zu. Im Sommer des Jahres 1958 hatte der Wissenschaftsrat Ausschüsse eingerichtet, in denen die Situation an den Medizinischen Fakultäten in Bezug auf „Vermassung“, „Spezialisierung“, „Forschungsrückstand“ und „hierarchische Strukturen“ problematisiert wurde. Diese Topoi waren seit dem Streit über die „Krise der Medizin“ in der Weimarer Republik vertraut und wurden auch in der Nachkriegszeit intensiv diskutiert (Geiger 2010). Die Ausschüsse setzten sich vor allem aus Professoren der Inneren Medizin sowie Verwaltungsfachleuten der Länder und des Bundes zusammen.^1^ Um zugleich mehr Studienplätze zu schaffen, eine praxisnahe Ausbildung zu ermöglichen, den oft beklagten Forschungsrückstand aufzuholen und trotz naturwissenschaftlich-technischer Spezialisierung die Einheit der Universitätsmedizin zu bewahren, erschien es notwendig, die starren Strukturen an den Fakultäten aufzubrechen.

Dezidiert ausformuliert wurden diese Planungen in den *Empfehlungen des Wissenschaftsrates zum Ausbau der wissenschaftlichen Einrichtungen* im November 1960 (Wissenschaftsrat 1960). Im Bereich der Medizin wurde den bestehenden Fakultäten dabei die Kompetenz zur Veränderung abgesprochen. Deshalb wurde ein Jahr später vom Wissenschaftsrat die Neugründung von sieben Medizinischen Akademien ausgeschrieben, in denen eine Studienreform mit Teamwork und Departmentsystem verbunden werden sollte. Der Mittelbau sollte dabei als Medium für die Integration der „Spezialisten“ in die Universitätsklinik fungieren. Vom Wissenschaftsrat wurde deshalb die Einrichtung von Dauerstellen für relativ selbstständige „Abteilungsvorsteher“ gefordert.^2^

Entscheidend für das Gelingen der Hochschulreform und die Lösung des Problems der Spezialisierung war also die „Frage des personellen Mittelbaues“, wie der Anatom Wolfgang Bargmann, von 1961 bis 1964 Leiter der wissenschaftlichen Kommission des Wissenschaftsrats, es konkret ausdrückte.^3^ Der Internist Fritz Hartmann, der sich in den Ausschüssen für die Neugründung von Medizinischen Akademien einsetzte, sprach jedoch ironisch davon, dass aktuell der Mittelbau gegenüber den Ordinarien ein „stabiles Subordinationsverhältnis“ habe.^4^ Der Psychosomatiker Thure von Uexküll votierte entsprechend für einen gleichberechtigten Status des Mittelbaus. Ein Organisationsschema mit einem Ordinarius als *primus inter pares* würde am ehesten die Wahrung der Einheit der Hochschulmedizin gewährleisten.^5^ Dies hatte der Kölner Physiologe Max Schneider 1957 ähnlich vorgeschlagen: Der Ordinarius sei mit einer viel zu großen Vormachtstellung ausgestattet, sodass er oft genug nicht nur sachlich, sondern auch menschlich überfordert sei. Wenn das Ordinariat als allein erstrebenswertes Ziel des „akademischen Nachwuchses“ erscheine, müsse dessen Nichterreichen auch fälschlicherweise als Scheitern aufgefasst werden. Dem Ordinarius müsse die „Baronie“ genommen werden (Schneider 1957: 1119).

## Das Projekt: Die Auflösung hierarchischer Strukturen

Mitte der 1960er Jahre sollten allerdings nur noch an drei Orten diese Planungen in die Tat umgesetzt werden: 1964 in Lübeck, 1965 in Hannover und 1967 in Ulm. Die Bezeichnung „Akademie“ wurde ausschließlich und auch nur bis 1973 in Lübeck verwendet. Da in diesen Institutionen sowohl klinisch als auch vorklinisch gelehrt wurde, unterschieden sie sich von der bereits seit 1923 bestehenden, aber klinisch orientierten Medizinischen Akademie Düsseldorf. Durch ihre explizite Ausrichtung nach dem US-amerikanischen Modell wurden sie auch implizit von den zeitgleichen Akademiegründungen in der DDR abgegrenzt. Die bei Planungen vorgebrachte Programmatik umfasste die drei zentralen Themen der Studienreform, der Stärkung des Mittelbaus und der Vertikalisierung der Hochschulstruktur. So diktierte auch Max Motz vom Bundesministerium für wissenschaftliche Forschung seinem Chef Gerhard Stoltenberg (CDU), dass in Ulm eine Universität errichtet werde, die in ihrer Konzeption etwas völlig Neues darstelle. Dies umfasse die Verbindung von Naturwissenschaften und Medizin, Teamwork statt Direktorialprinzip, die Gleichberechtigung des Mittelbaus und die Neugestaltung des Studiums nach den Empfehlungen des Wissenschaftsrates.^6^ In Pressemeldungen zu den Planungen in Hannover hieß es, dass dort dem Mittelbau im Departmentsystem eine eigenständige Position zukommen solle (St. 1965). Unabdingbar für die Integration eines selbst integrierend wirkenden Mittelbaus waren dabei Festanstellungen und die verfassungsmäßige Gleichstellung. Uexküll stellte dazu kategorisch fest, dass nur so überhaupt Kontinuität an den spezialisierten Universitätskliniken ermöglicht werden könne (von Uexküll 1962: 24).

Das Departmentsystem konnte also als Anpassung an die Erfordernisse einer modernen und effektiven Universität verstanden werden, war aber auch als Modell zu deren Demokratisierung gedacht. Dafür brauchte es jedoch eine fundamentale Neugestaltung der Hochschulen. Gemäß den Strukturplänen in Hannover und Ulm sollte die große Einheit der Fakultät in „Zentren“, „Abteilungen“ und „Sektionen“ aufgelöst werden. Der Kitt dieser Restrukturierung war der Mittelbau. Der Abbau der Fakultäten und ein selbstständiger Mittelbau bedingten einander. Dies musste zugleich Auswirkungen auf das personelle Gefüge haben. Das direktoriale System sollte explizit durch ein Kollegialsystem ersetzt werden. Die innere Struktur der Neugründungen, so drückte es der Internist und Gründungsrektor der Medizinischen Hochschule Hannover Rudolf Schoen unmissverständlich aus, beruhe auf der Auflösung „der strengen klassischen hierarchischen Struktur“.^7^

## Das Scheitern: Der Widerstand der Ordinarien

Die Umsetzung dieser Hochschulplanungen gestaltete sich jedoch aus unterschiedlichen Gründen schwierig. Besonders hemmend wirkten sich die seit 1966 stark gedrosselte Finanzierung seitens der Länder und der Widerstand der Ordinarien aus. Letzterer vor allem, als um 1970 in der Öffentlichkeit die Revolte der Studierenden mit den Hochschulreformplanungen identifiziert wurde (Rohstock 2010: 381f.). Die Zerstreuung der „Studentenmassen“ in der Medizinausbildung war um 1960 ein wichtiges Ziel der Empfehlungen des Wissenschaftsrats gewesen. Dies sollte durch mehr Ausbildungsstätten erreicht werden. Allerdings stieß die Erhöhung der Kapazitäten rasch an ihre Grenzen – vor allem, weil es schlicht zu weniger Neugründungen kam als vorgesehen. Zahlreiche Medizinische Fakultäten verließen sich stattdessen auf das Mittel des Numerus clausus. Da dieses aber einem Urteil des Bundesverfassungsgerichts im Sommer 1972 nicht standhielt und die Hochschulen gemäß der daraus folgenden Kapazitätsverordnung deutlich mehr Studierende aufzunehmen hatten, gerieten die Reformpläne in die Krise. Auch mit der Ende 1970 verkündeten und im Oktober 1972 vollständig in Kraft getretenen Approbationsordnung wurden zwar vertikale Fächer wie Psychosomatik, Sozialmedizin und medizinische Psychologie in die Medizinausbildung integriert, aber weitere Reformideen nicht verbindlich festgelegt (Forsbach 2011: 116f.; Schagen 2002: 10–15). Vor allem verschärfte sich der Disput über die Frage der Mitbestimmung in Forschung und Lehre: Konnte die Ordinarienuniversität durch ein Departmentsystem abgelöst werden, bei dem „die Professoren“ nicht länger die Mehrheit hätten?

Die Ordinarien gerieten dabei öffentlich unter Druck. Der Wissenschaftsrat bestätigte 1968 noch einmal, dass es weiterhin um die Abschaffung des hierarchischen Systems der Ordinarienuniversität gehe (Wissenschaftsrat 1968). Ein Jahr später machte auch der *Spiegel* die „totale[n] Ordinarien-Herrschaft in den medizinischen Ausbildungs- und Forschungsstätten“ als Hauptproblem aus. Die medizinischen Fakultäten hätten sich als handlungsunfähig gegenüber notwendigen Neuerungen erwiesen. Die „ganze Ordinarien-Universität mit ihren Petrefakten“ müsse aufgegeben und der akademische Mittelbau aus uralten Abhängigkeiten gelöst werden (Hentschel 1970: 21, 53).

Die Ordinarien auf der anderen Seite blieben nicht tatenlos. Der Westdeutsche Medizinische Fakultätentag war bereits angesichts der Gründungspläne für Medizinische Akademien alarmiert gewesen, hatte in einer Stellungnahme die Beibehaltung eines streng gegliederten Systems in der Klinik gefordert und die Empfehlungen des Wissenschaftsrats abgelehnt.^8^ Mit dem „Marburger Manifest“ sprachen sich im April 1968 große Teile des fast ausschließlich männlichen akademischen Establishments, welche die „Freiheit von Lehre und Forschung“ in höchster Gefahr sahen, öffentlich gegen die Mitbestimmung der Studierenden und des Mittelbaus aus (Koischwitz 2017: 81–93).

Die erhitzten Diskussionen über drittel- oder viertelparitätische Mitbestimmung in den Hochschulgremien, die in einigen Bundesländern bereits auch in Gesetzesform gebracht worden waren, wurden im Mai 1973 abrupt gestoppt, nachdem das Bundesverfassungsgericht entschieden hatte, dass die Mitbestimmung der nun so bezeichneten „Hochschulgruppen“ zwar zulässig sei, bei Entscheidungen zu Forschung und Lehre, insbesondere auch bei Berufungen, aber die „Professorenmehrheit“ gesichert sein müsse. Die Gruppe der „Hochschullehrer“ sollte zudem explizit homogen zusammengesetzt sein. Das entscheidende Kriterium dafür war die Habilitation. Indem die hegemoniale Stellung der Ordinarien in Forschung und Lehre schließlich in dem 1976 in Kraft getretenen Hochschulrahmengesetz festgeschrieben wurde, wurden auch die reformerischen Aspekte mit Ausnahme der Studienreform wieder eingehegt. Während in den Planungen um 1960 der Mittelbau als Lösung der Krise nicht nur der Medizin, sondern auch der Hochschulen selbst angesehen wurde, erschien er seit 1970 auf nachhaltige Weise wieder als ein Problem, das mit Steuerungsmaßnahmen reguliert werden musste.

## Die Bilanz: Der Mittelbau unter den Rädern

Die als Akademieprojekte gestarteten Neugründungen waren von diesen Entscheidungen unterschiedlich betroffen. In Ulm war die Departmentstruktur bereits 1972 unter Beschuss geraten. Als der Humangenetiker Helmut Baitsch am 5. Dezember 1974 seinen Rücktritt als Rektor der Universität Ulm einreichte, erklärte er dies vor allem mit dem „Ersticken praktisch aller Reformansätze in der rigorosen Mangelsituation“. Für Baitsch stand fest, dass es sich beim grandiosen Reformvorhaben der Universität Ulm, das einmal die internationale Fachwelt begeistert hatte, bereits wieder um ein abgeschlossenes Kapitel handelte.^9^ 1985 identifizierte der Angiologe Klaus Alexander, zu dieser Zeit Rektor der Medizinischen Hochschule Hannover, die Hochschulgesetzgebungen der frühen 1970er Jahre schlicht als Ende der Reformprojekte (Alexander 1985).

Unter die Räder gekommen war auf jeden Fall der Mittelbau, den der Soziologe Burckhardt Kaddatz 1986 bereits treffend als „Projektpersonal“ klassifizierte (Kaddatz 1986). Innerhalb von zwanzig Jahren waren die Pläne des Wissenschaftsrats ad acta gelegt und das alte Ordinariensystem als zugleich differenzierte und hierarchisierte Gruppenhochschule reetabliert worden. Ein in Departments organisierter Mittelbau wurde nicht zum Zentrum moderner und demokratischer Hochschulen, sondern wird seitdem wieder als Provisorium auf dem Weg zur Professur verstanden, bei dem Promovierte um rare Stellen konkurrieren. Das Ende der „Baronie“ ist auch im 21. Jahrhundert noch keineswegs abzusehen.

## Footnotes

1 Der Wissenschaftsrat bestand bis 1965 ausschließlich aus Männern. Siehe Bartz (2007: 59).

2 „Protokoll über die 11. Sitzung der Vollversammlung des Wissenschaftsrates am 10. Juni 1961 in Berlin“ (Landesarchiv Schleswig-Holstein, Abt. 811, Nr. 20919 I).

3 „Protokoll über die 2. Sitzung des Unterausschusses ‚Medizinische Akademien‘ am 16.02.1961 in Köln“ (Bundesarchiv Koblenz, B/247, 16).

4 „Protokoll der 23. Sitzung des Ausschusses ‚Medizinische Akademie Hannover‘, 12./13.11.1964“ (Archiv der MHH, GRÜA 10020 2).

5 „Protokoll über die 6. Sitzung des Unterausschusses ‚Medizinische Akademien‘ am 18.10.1961 in Köln“ (Bundesarchiv Koblenz, B/247, 16).

6 Max Motz, „Sprechzettel zur IMA-Sitzung vom 30. Juni 1966“, 23.06.1966 (Bundesarchiv Koblenz, B/138, 6509).

7 „Protokoll der 14. Sitzung des „Ausschusses Medizinische Akademie“ in Hannover, 02./03.09.1963“ (Archiv der MHH, GRÜA 10019 1, E 2.1, Nr. 1).

8 „Stellungnahme des Medizinischen Fakultätentages zu den Anregungen des Wissenschaftsrates zur Gestalt neuer Hochschulen und zu den Empfehlungen zur Entlastung der medizinischen Fakultäten“, o. D. (Archiv der MHH, G Sammlung Schneider, Überlegungen des Wissenschaftsrates zur Medizin, 1951–1963. Band 2).

9 H. Baitsch, „Rechenschaftsbericht für die Zeit vom 01.01.1974–31.03.1975“ (Archiv der MHH, Dep. 1, acc.2011/Nr. 10).
